# Pharmacological Activities of Aminophenoxazinones

**DOI:** 10.3390/molecules26113453

**Published:** 2021-06-07

**Authors:** Jesús G. Zorrilla, Carlos Rial, Daniel Cabrera, José M. G. Molinillo, Rosa M. Varela, Francisco A. Macías

**Affiliations:** Allelopathy Group, Department of Organic Chemistry, Institute of Biomolecules (INBIO), Campus CEIA3, School of Science, University of Cadiz, 11510 Puerto Real, Cádiz, Spain; jesus.zorrilla@uca.es (J.G.Z.); carlos.rial@uca.es (C.R.); danielcabreratorrejon@hotmail.com (D.C.); chema.gonzalez@uca.es (J.M.G.M.); rosa.varela@uca.es (R.M.V.)

**Keywords:** aminophenoxazinone, drug, Phx-3, cytotoxicity, cancer, antibacterial, antifungal, virus, in vitro, in vivo

## Abstract

Aminophenoxazinones are degradation products resulting from the metabolism of different plant species, which comprise a family of natural products well known for their pharmacological activities. This review provides an overview of the pharmacological properties and applications proved by these compounds and their structural derivatives during 2000–2021. The bibliography was selected according to our purpose from the references obtained in a SciFinder database search for the Phx-3 structure (the base molecule of the aminophenoxazinones). Compounds Phx-1 and Phx-3 are among the most studied, especially as anticancer drugs for the treatment of gastric and colon cancer, glioblastoma and melanoma, among others types of relevant cancers. The main information available in the literature about their mechanisms is also described. Similarly, antibacterial, antifungal, antiviral and antiparasitic activities are presented, including species related directly or indirectly to significant diseases. Therefore, we present diverse compounds based on aminophenoxazinones with high potential as drugs, considering their levels of activity and few adverse effects.

## 1. Introduction

Secondary metabolites are natural products produced by organisms to participate in their biological processes. As they are the result of natural selection through evolution, they are highly optimized structures. Thus, their affinity with the proteins to be synthesized and modified, or their influence on pathological processes (most of the pharmacological targets are proteins), have made of these natural products an increasingly choice for the development of new drugs, either by themselves, or more frequently, through structural synthetic derivatives. These substances are excellent lead compounds in terms of selectivity, specificity, effectiveness and pharmacokinetics. Besides, their structural diversity allow one to secure suitable chemotheques [1].

Aminophenoxazinones are degradation products resulting from the metabolism of different plant species. They comprise a family of natural products well known for their pharmacological activities. In fact, this particularly high interest on these compounds started more than a century ago, as their different industrial and medical applications as pigments, pesticides or drugs were being discovered. Aminophenoxazinones are tricyclic structures with double bonds in aromatic systems containing oxygen and nitrogen atom, which facilitates the development of synthetic derivatives to enhance the properties of these molecules.

This review provides an overview of the research works that have focused on the pharmacological properties and applications of aminophenoxazinones and its structural derivatives. The most relevant previous review related to aminophenoxazinones was published in 2013 and was focused on Phx-3 [2]. The bibliography was selected using the SciFinder database, by seeking articles related to the 2-aminophenoxazine-3-one (Phx-3) structure, the base structure of all aminophenoxazinones. The search was restricted to 2000–2021. The resulting articles (284) were subjected to a critical reading to select those providing information on the pharmacological activities of both aminophenoxazinones and their structural derivatives. Besides, some complementary searches were carried out through SciFinder or Google Scholar to complete this review. Thus, we provide herein an updated overview, as well as the description of other aminophenoxazinones and derivatives.

First, we provide information about the biosynthesis of aminophenoxazinones in plants. Then, their anticancer activities will be presented, including the most relevant results that have allowed us to expand knowledge about their mechanism(s) of action. Antibacterial, antiviral and antiparasitic properties are subsequently detailed. To quantify the levels of activity and facilitate a comparison of the compounds, the values of the half maximal inhibitory concentrations (IC_50_) provided in the bibliography for the different assays have been included. The compounds named Phx-1, Phx-2 and Phx-3 have been the most evaluated in the literature, especially Phx-3, having shown a number of promising properties.

## 2. Aminophenoxazinones as Degradation Products of Benzoxazinones

**Figure 1:**
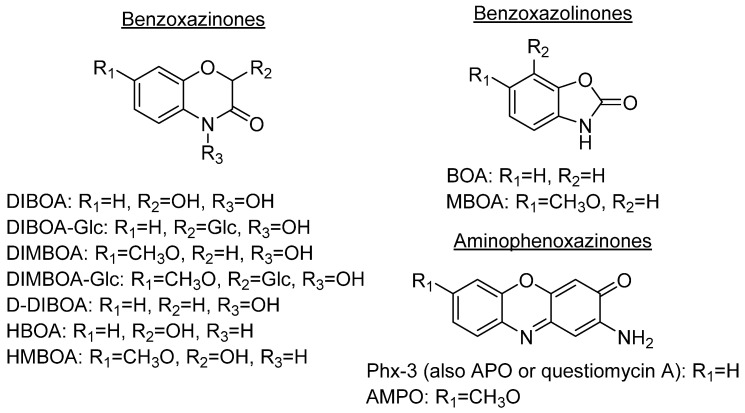
Prominent benzoxazinones and some of their degradation products.

Some plant species, mainly those from the Gramineae family, which comprises prominent crops such as wheat, rye or maize, produce benzoxazinones like the cyclic hydroxamic acids 2,4-dihydroxy-1,4-benzoxazolin-3-one (DIBOA) and 2,4-dihydroxy-7-methoxy-1,4-benzoxazin-3-one (DIMBOA) (Figure 1). Those, together with different degradation metabolites, are well known for their biological activities [3,4]. In fact, the potential benefits of the intake of food containing benzoxazinones would be in agreement with the time that they remain inside the human body, with dose-dependent absorption (peak in plasma after 3 h) and rapid washout [5]. Some examples of these highly interesting compounds with these structures are 2-hydroxy-1,4-benzoxazin-3-one (HBOA), which is proposed as the biosynthetic precursor of DIBOA and DIMBOA [6], as well as 2-hydroxy-7-methoxy-1,4-benzoxazin-3-one (HMBOA), a degradation product of DIMBOA exuded by wheat [7]. A study of a diet based on rye bread revealed that the 2-β glycosylated derivatives of HBOA and DIBOA are the major benzoxazinoids in plasma and urine [8]. The concentrations measured for all the detected compounds in this study would provide key information for understanding their bioavailability, absorption and metabolism. In fact, DIBOA has shown inhibitory effects on the growth of cancer lines like DU145 (prostate), which motivated the development of in vivo studies on human and the intake of enriched food [9].

Because of the diversity of products that can be obtained from the degradation of hydroxylated benzoxazinones depending on their cultivation soil [10] or the extraction method, which could be either by submerging them into aqueous solutions [11] or by putting them in contact with certain microorganisms [12], they have always been a focus of interest.

**Scheme 1:**
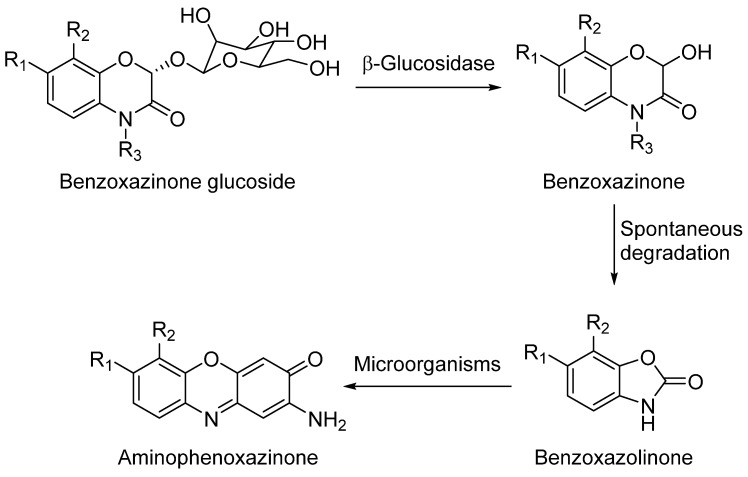
General degradation process of benzoxazinones.

DIBOA and DIMBOA can be detected inside plants as their β-D-glucosides DIBOA-Glc and DIMBOA-Glc. β-D-Glucosides are transformed into DIBOA and DIMBOA when plants are damaged, by enzymatic mechanisms catalyzed by a β-glucosidase (Scheme 1). The latter compound has also been detected as the major benzoxazinone in *Triticum aestivum* L., whereas DIBOA-Glc is mainly concentrated in *S. cereale* L. [13]. DIMBOA is unstable in soil and spontaneously transforms into 6-methoxybenzoxazolin-2-one (MBOA), and subsequently turns into 2-amino-7-methoxy-3*H*-phenoxazin-3-one (AMPO) through the action of edaphic microorganisms when submerged into aqueous solutions [14]. This fact could explain the higher resistance of AMPO to biodegradation. A similar case occurs with the analogous DIBOA and its benzoxazin-2(3*H*)-one (BOA) that turns into to Phx-3 (also known as APO or questiomycin A). However, DIBOA has a high persistence and could have a more important role in chemical defence mechanisms than DIMBOA in plants that produce large amounts of hydroxamic acids [11].

In comparison to their precursors with benzoxazinone structures, aminophenoxazinones like Phx-3 are much stable and easier to extract. These advantages, which have enabled the development of numerous studies on their biological activities [13] and modes of action [3] have also facilitated their production for commercial purposes. In the case of Phx-3, Venturelli et al. demonstrated that its phytotoxic effects against the growth of certain plants (like the model species *Arabidopsis thaliana*) are related to the inhibition of histone deacetylases, which provokes locus-specific alterations in histone acetylation processes [15]. In certain agronomical contexts, some studies like this or the conducted by Macías et al. reflect how lower concentrations of Phx-3 successfully achieve high phytotoxicity levels [16]. Another interesting phytotoxic-related compound is D-DIBOA, which has been proposed as an excellent candidate for the development of new herbicides [17].

The degradation process of benzoxazinones functionalized with hydroxamic acid groups leads to benzoxazolinones [11]. BOA and DIBOA, from benzoxacinone-producer plants like maize [18], wheat [19] or rye [20] are the most representative isolated compounds obtained until now.

Aminophenoxazines are an interesting family of tricyclic compounds related to the previously mentioned benzoxazinones and benzoxazolinones. They are originated as a result of the decomposition of benzoxazolinones into their corresponding 2-aminophenol and its subsequent dimerization.

A wide variety of aminophenoxazines has been developed because of their pharmacological interest (and also agronomical). Their antiviral [21], antitumoral [22] and antibiotic [23] activities have been studied by those methods that apply the dimerization of two phenolic units into a tricyclic compound. A process that depends not only on the phenol itself, but also on the reaction conditions. In the case of Phx-3, its dimerization was accomplished through a hydrometanolic solution [24] combined with the use of photochemistry [25]. Different aminophenoxazines have been obtained by applying this method to alkyl, halo or nitroaminophenols.

**Figure 2:**
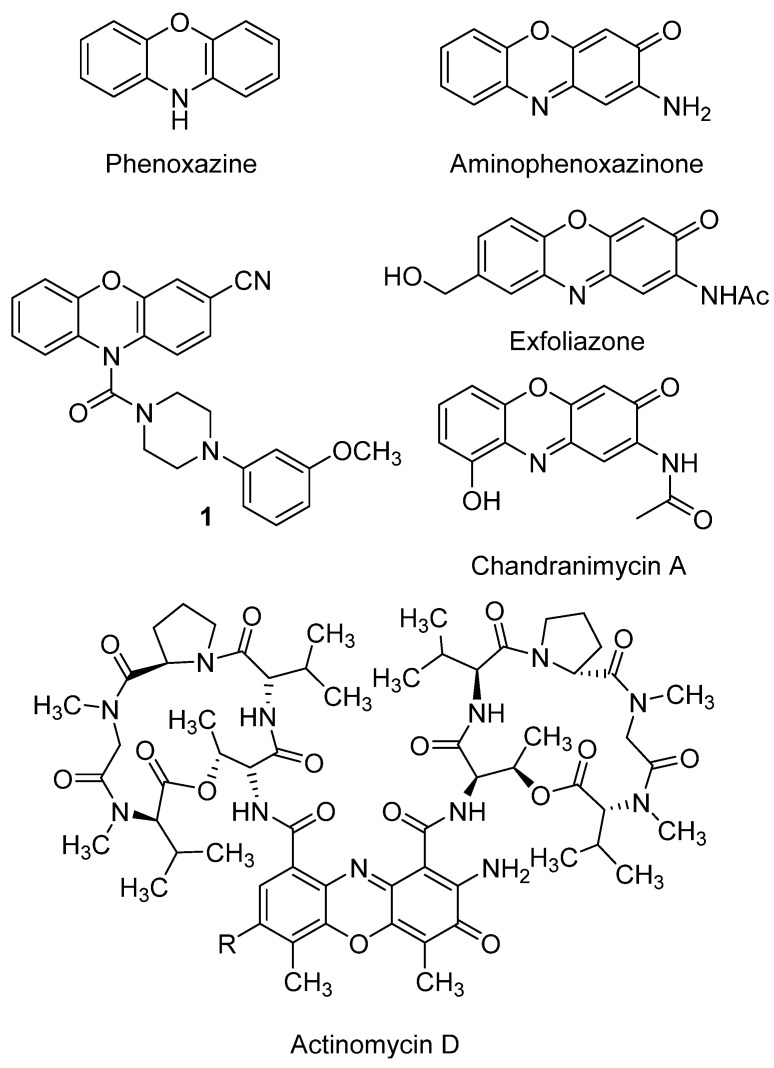
Phenoxazine and aminophenoxazinone structures, and some molecules of their different derivatives, like the drug actinomycin D.

The chemistry of phenoxazines (Figure 2), the scaffold of aminophenoxazines, has attracted the interest of researchers for more than a century because of their wide range of applications in the manufacturing industry and in medicine. Phenoxazine was first prepared in 1887 by thermal condensation of *o*-aminophenol with catechol [26]. Since then, several studies have developed new derivatives of phenoxazine, proving their effectiveness as drug compounds. The study of Prinz et al. is a recent example, where a new family of *N*-heterocyclic (4-phenyl-piperazin-1-yl)methanones derivatives showed excellent inhibition growth activities against different tumor cell lines, being remarked the activity levels of compound **1** (Figure 2) [27].

It is worth highlighting the great number of articles published in recent years about new phenoxazine derivatives with applicability for detecting specific compounds related to diseases. For example, new fluorophores containing two molecules of phenoxazine (or phenothiazine, where the oxygen atom is replaced by a sulfur one) have been developed for in vivo tumor detection by formation of fluorescent polymer nanoparticles [28]. Similarly, BC-2 and BC-3 are compounds based on a phenoxazine scaffold, which have been successfully tested in vivo to detect hypochlorous acid, compound whose overproduction is related with diseases like atherosclerosis or rheumatoid arthritis [29].

Aminophenoxazines (Figure 2) were first applied as industrial colorants and pigments. Further properties and their corresponding applications such as antioxidants [30], polymerization stabilizers, pesticides, insecticides [31], biological markers [32], acid-base indicators [33] and, with more relevance, drugs [31] were gradually found.

The increasing number of the studies that demonstrated the pharmacological properties of aminophenoxazines made the main focus of attention to move from its use as a dye to the biological field. In fact, these tricyclic compounds are the structural base of several pharmacotherapeutic agents with a wide range of biological properties.

A recent study on the biological activities of certain synthetic aminophenoxazinones rendered highly promising results regarding the development of new drugs with antibacterial, antifungal, anticancer, reversal resistance as well as anti-inflammatory, antiviral or antifilarials [31]. Moreover, aminophenoxazine is the base of actinomycin D, a well-known antibiotic obtained from *Streptomyces antibioticus* [34]. This antibiotic can present potent high anticancer activity, but also has some side effects that must be taken into account, such as myelosuppression or renal cytotoxicity [35]. Actinomycin D currently continues being studied. As an example of recent positive result, the increase of effectiveness for ovarian cancer treatment found for its synergic use with photodynamic therapy can be mentioned [36].

The study of structure-activity relationships (SAR) makes possible to define optimal structural characteristics to improve the properties of the molecules. That is the case of the natural product chandrananimycin A (Figure 2), whose hydroxyl group was related with the better cytotoxicity levels of the compound against HeLa cell line (IC_50_ = 8.87 ± 3.49 µM) [37]. A second example of SAR is the conducted by Pasceri et al. which proved that the electron-withdrawing carboxylate group is associated to a moderate activity of a new a family of synthetized Phx-3 derivatives (including the natural products chandrananimycin A and exfoliazone, Figure 2) on the inhibition on indoleamine 2,3-dioxygenase (an enzyme that is associated to the suppression of the immune response in some forms of cancer) [38]. More in depth studies focused on SAR are required to provide firm conclusions, being usual that the SAR conclusions provided in the bibliography might be supported by additional studies which evaluate a wider number of related structural derivatives.

## 3. Anticancer Activity

**Figure 3:**
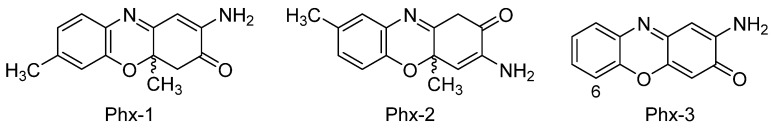
Most studied aminophenoxazinones: 2-amino-4,4*α*-dihydro-4*α*,7-dimethyl-3*H*-phenoxazin-3-one (Phx-1), 3-amino-1,4*α*-dihydro-4*α*,8-dimethyl-2*H*-phenoxazin-2-one (Phx-2) and 2-aminophenoxazine-3-one (Phx-3).

In general terms, cancer is an uncontrolled cellular division process that provokes the aggregation of cells and, subsequently, the formation of tumors. A number of additional factors such as genetic mutations, improper diet, smoking or the ingestion of heavy metals and other contaminants may also have some influence on this process by altering the signaling pathways [39,40]. The following sections explain in detail the effect of aminophenoxazinones on different types of cancer. The compounds Phx-1, Phx-2 and Phx-3 (Figure 3) have been the most studied aminophenoxazines. Their use as anticancer drug aims at the generation of apoptosis of cancer cells, by altering or generating specific mechanisms.

### 3.1. Gastric and Colon Cancer

Gastric cancer is the third cause of cancer-related death worldwide and a common pathology with a high incidence rate [41], being nowadays a great clinical challenge. Three treatment methods are commonly performed: surgery, endoscopic resection and chemotherapy [42]. Unfortunately, these treatments do not guarantee a satisfactory level of effectiveness, even if their results are rather promising, for example due to the risk of metastasis. On the other hand, the use of different types of synthetic drugs have shown unpredictable side effects. Despite the advances in the understanding of the pathophysiological mechanisms and in the treatment of gastric cancer in recent years, no significant changes have been achieved yet [43] because of no significant alternatives have been established, especially for patients with delayed diagnoses [44].

The intracellular pH (pHi) varies in accordance with the acidification of the extracellular medium as homeostatic response. However, cancer cells do not decrease this pHi as expected, despite of tumors lead to the acidification of the extracellular pH. The regulatory system commonly concluded to be related is the membrane protein Na^+^/H^+^ exchanger called NHE-1 [45]. Several studies have suggested that a decrease in pHi may precede apoptotic events in cancer cells. Therefore, drugs with ability to induce these reductions are considered optimal candidates to treat cancer by triggering programmed death of the cells [46]. Some examples of such bioactive compounds are etoposide, cycloheximide or camptothecin. However, the three of them have significant adverse effects on the host [47,48].

2-Aminophenoxazin-3-one (Phx-3) in particular, stands out according to the numerous studies that have proven its effectiveness to change the pHi of different cancer cell lines [49]. In the study of Che et al., the treatment of KB-31 (squamous carcinoma) and K562 (chronic myeloid leukemia) human cell lines (both with a pHi much higher than normal cells) with Phx-3 significantly decreased the pHi (up to 0.9 units) after 20 min. Phx-1 also generated a pHi decrease, but less marked. Authors have compared the activity of both aminophenoxazinones with that of camptothecin, being this a positive result due to the low or null adverse effects found for Phx-1 or Phx-3 in different studies [50,51]. However, the mechanism that causes the pHi decrease in these cells lines when Phx-3 or Phx-1 are administrated is not clear. Therefore, it becomes necessary to determine if a pHi decrease precedes the apoptosis of cancer cells and its relation with the activity of NHE-1.

Kasuga et al. [52] determined that the growth of the gastric cancer cells MKN45 and KATO III is inhibited by treatment with Phx-1 or Phx-3, whereas the apoptotic mechanism would be associated with a caspase-independent pathway. The changes of pHi caused by Phx-3 in gastric cancer cells were not investigated until 2011 [53], when the effect of Phx-3, associated with NHE-1, on gastric cancer cells was investigated in terms of how they affected pHi, as well as with regard to the possible causal correlation between a decrease in pHi and the appearance apoptotic events. The method of Litman et al. [54] was used to estimate the pHi values by fluorescence. The results confirmed that the treatment of cancer cells with Phx-3 produces a rapid decrease in pHi depending on the dose administered: by 1.65 units for MKN45 cells and by 1.3 units for MKN74 cells after the administration of a 100 µM solution of Phx-3. This dose also suppressed NHE-1. These results suggested that Phx-3 provokes a decrease in pHi that precedes the apoptotic events, whereas the mechanism for inhibiting NHE-1 was associated with the interaction of Phx-3 with the lipid layers by altering the lipid properties of the cells’ membranes. Moreover, Phx-3 causes a significant depolarization of mitochondria, which activates caspases and influences apoptotic events in cancer cells. For further insight on Phx-3 mode of action against gastric cancer (as well as against colon cancer, presented hereunder), please, see the review by Tomoda et al. [2].

Colorectal cancer (colon and rectal altogether cancer) is the third most frequent cancer and the second leading overall cause of cancer deaths [41]. The in vitro activity of Phx-3 in human colon cancer cell lines and specifically on COLO201, DLDI and PMCO1 lines has been determined [55]. The results demonstrated that this aminophenoxazinone causes high levels of cytotoxicity at low doses (IC_50_ value between 6–12 μM) and pro-apoptotic effects in these lines. Phx-3 also generates cytotoxic effects on HT-29 (IC_50_ = 16.7 μM) [56] and LoVo-1 lines (IC_50_ = 20.03 ± 4.98 μM) [57]. In the same studies, Phx-1 has also shown activity against all colon cancer lines mentioned, although to a lesser extent.

The review of Tomoda et al. [2] gathered information about indirect anticancer activity of Phx-3, being worth highlighting at this point the high suppressive activity found in vitro [58] to generate the anion superoxide in neutrophils. The overproduction of this anion is related with ulcerative colitis, a disease associated with colon cancer.

Thus, studies like the ones detailed in this section support that Phx-3 would be an accurate agent itself to treat gastric and colon cancers. Regarding in vivo evaluations of adverse effects, experiments in which mice were treated with (orally administered) Phx-3 showed that, in addition, this compound causes low adverse effects, since they did not show gastrointestinal lesions or diarrhea during 4 weeks of administration [59].

**Figure 4:**
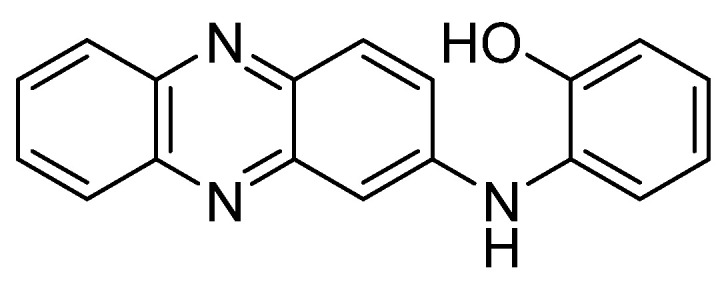
Structure of *N*-(2-hydroxyphenyl)-2-phenazinamine (NHP).

Even though the Phx family (mainly Phx-3) has been the most studied, it is possible to find evaluations of other derivatives. This is the case of *N*-(2-hydroxyphenyl)-2-phenazinamine, known as NHP (Figure 4), a secondary metabolite isolated for the first time in 2012 from a marine actinomycete [60]. This molecule possesses the tricyclic scaffold of Phx-3, bonded to a fourth aromatic ring (a phenol). Besides, in comparison to Phx-3, NHP contains an additional nitrogen atom in the second ring (in substitution of the oxygen), and lacks of the carbonyl group. This new molecule showed high cytotoxic activity against the colon adenocarcinoma line HCT116 (IC_50_ = 27.82 µg/mL). Moreover, NHP was active against other human lines: COC1 (ovarian cancer, IC_50_ = 28.11 µg/mL), HepG2 (liver hepatocellular carcinoma, IC_50_ = 40.33 µg/mL) and A549 (lung adenocarcinoma, IC_50_ = 38.53 µg/mL). At comparing the activity of NHP with that found for Phx-3 against two of these lines in other studies, it is worth highlighting that the corresponding IC_50_ value of NHP is significantly higher than that shown by Phx-3 against A549 (5.48 ± 0.38 µg/mL), whereas a value of 6.58 ± 0.61 µM was obtained for the HepG2 line. On the other hand, the value of NHP against A549 improves that of the aminophenoxazinone Phx-1 (78.29 ± 5.11 µg/mL) [57,61]. The comparison of these results with the structure of the three compounds (NHP, Phx-1 and Phx-3) suggests that a deep structural-activity relationships study would be necessary to determine which structural characteristics provokes the differences between the mentioned activity levels.

### 3.2. Glioblastoma

Glioblastoma is the most common type of aggressive brain tumor in adults, whose treatment is considered a high challenge for oncology since surgery, chemotherapy or radiotherapy are not enough to prevent the tumor progression. One of the main difficulties is achieving the cross of sufficient doses of the chemotherapeutic agents through the blood-brain barrier [62]. The ability of some phenoxazine and phenothiazine derivatives to cross this blood-brain barrier was discovered [63]. This represents an invaluable potential reason for these compounds to be used as anticancer drugs for the treatment of glioblastoma. The anti-tumor activity of phenothiazines was lower, though it should be noted the effectiveness of these compounds as drugs for the treatment of psychotic disorders [64].

Glioblastoma is a complex disorder that appears as a response to a particular form of cellular damage by which cells’ signaling pathways get altered. Cell differentiation is then affected, and as different cell growth factors are over-expressed, the glioblastoma cells get activated and the oncogenesis process, the process by which a normal cell becomes a carcinogen, is promoted [65]. Two of the signaling pathways most related to growth factors, and along with to oncogenesis, are the phosphatidylinositol 3 kinase (PI3K/AKT) and the RAS/RAF/MAPK (ERK) [66]. AKT is a protein kinase, frequently hyperactivated in tumors, with the capacity to deactivate certain proapoptotic factors (like caspase-9) while favoring the expression of anti-apoptotic genes. Moreover, it deactivates certain tumor suppressor genes through phosphorylations, which makes glioblastoma tumors susceptible to AKT [67]. In the case of ERK, its activation is associated with cell survival, proliferation, and death [68]. The protein c-jun N-terminal quinase (JNK) is also involved in pro-apoptotic. It is activated when subjected to different types of stress such as the exposure to anticancer drugs, osmotic stress or radiation [69].

**Figure 5:**
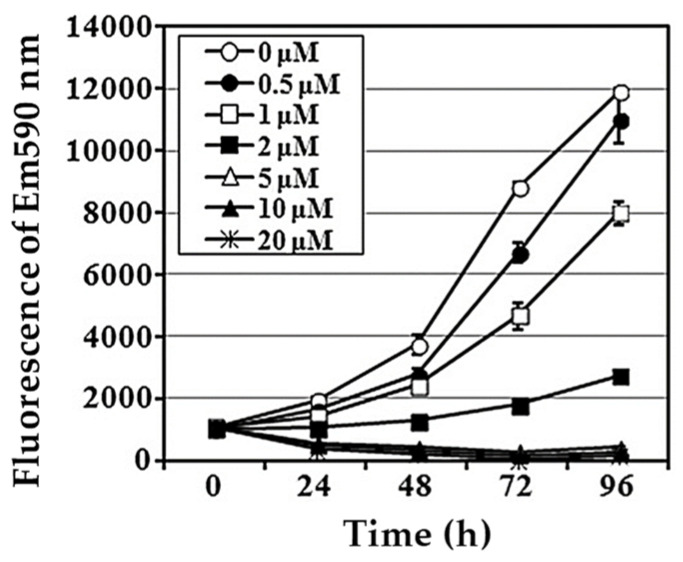
Inhibition of cell growth and induction of apoptosis after treating LN229 cell line with Phx-3. Source: Che et al. [70] by permission from Spandidos Publications Ltd.

Focusing on the aminophenoxazinone Phx-3, Che et al. [70] studied its apoptosis-inducing effect on LN229 glioblastome cell line. Figure 5 shows how the cell growth was significantly inhibited by Phx-3 at 1 µM, being strongly inhibited at concentrations above 2 µM. The IC_50_ values were 2.602 ± 0.087 µM (24 h) and 1.655 ± 0.093 µM (48 h), which confirms the potent inhibitory effect of Phx-3 at low concentrations against LN229 cell line, being one of the lowest values ever measured until 2013. As an example of the previous study on the effect of Phx-3 on glioblastoma cell lines, let us point out the remarkable cell growth inhibition confirmed for U251MG cell [71].

Che et al. also clarified the molecular mechanisms of the apoptosis induced by Phx-3, by attending to the ERK and JNK signaling pathways. The levels of these phosphorylated pathways increased significantly (6 h) after treatment with Phx-3, being maintained for 20 h. The activation of JNK was suggested to be mediated by the generation of reactive oxygen species (ROS). On the contrary, the phosphorylation of AKT and mTOR (protein kinase that acts as a signal transducer) were suppressed. All these effects would contribute with the apoptosis induced by Phx-3, and the relevance of their roles was studied by the evaluation of the activity of Phx-3 in the presence or absence of specific inhibitors. The results showed as the phosphorylation of ERK would not represent a major pathway for the induction of apoptosis, whereas AKT would act under ERK signaling. In the case of JNK, its activation would play a key role in the induction of apoptosis. Long exposure to Phx-3 would activate alternative apoptotic mechanisms by mitochondrial demoralization, like the inhibition of mTOR activity.

Previous studies confirmed that treatment of the lung adenocarcinoma cell line A549 with Phx-3 resulted in the depolarization of the mitochondria, causing a decrease of pHi and the generation of ROS [72]. The suppression of the pathway of interest, JNK, would significantly protect against the induction of apoptosis by ROS. Therefore, the generation of ROS by mitochondria in LN229 cells was also examined by Che et al. [70], in the presence or absence of ROS scavengers (melatonin or Nac). Fluorescence values increased after 3 h of exposure to Phx-3 in a dose-dependent manner, whereas the ROS production was significantly inhibited in the presence a scavenger. Melatonin almost completely prevented the induction of apoptosis by Phx-3, and deactivated the JNK pathway. Regarding the other pathways, the application of Phx-3 in presence of a ROS scavenger restored the phosphorylation state of ERK and AKT to the control levels, and inhibited the phosphorylation of mTOR.

In summary, all the information gathered indicates that Phx-3 depolarizes mitochondria and decreases pHi, which causes the generation of ROS in mitochondria. Then, ROS activates the JNK pathway, which is a signaling pathways highly related to glioblastoma, and also the main generator of apoptosis in cancer cells by caspase cascade. The long exposure to Phx-3 would activate alternative mechanisms.

### 3.3. Melanoma

The incidence of melanoma has increased at a higher rate than other solid tumors, being currently the third most common skin cancer. When at advanced stages it represents a serious threat when it spreads to internal organs, which is the major cause of death in skin-cancer patients. This disease may be due to different causes, including both genetic and environmental factors. Several factors have already been identified as significant influencers on both the incidence and the clinical and oncogenic characteristics of this disease. These factors are UV exposure, use of tanning beds, a family history of melanoma, and phenotypic characteristics of fair skin and hair color [73,74].

Phx-3 has been considered as effective for inhibiting the proliferation of malignant B16 melanoma cells in mice in vitro and in vivo, causing few adverse effects [51]. In the in vitro assay, B16 cells were transplanted into mice with the presence of Phx-3, and the inhibition of the proliferation of cells was achieved in a time- and dose-dependent manner. In presence of this aminophenoxazinone (concentration of 41.5 µM), the inhibition was almost 80% after 48 h, and over 90% after 72 h. Regarding the in vivo effects of Phx-3 on cells, no tumor formation was observed (cutaneous or subcutaneous) after the transplantation of melanoma cells into the flank of mice, by using a very low concentration of Phx-3 (0.5 mg/kg). Besides, this dose of Phx-3 did not increase the tumor size. The different effective concentrations between the in vitro and in vivo activities was suggested to be related with the in vivo synergy of Phx-3 with cytokines, proteins involved in cell signaling.

**Figure 6:**
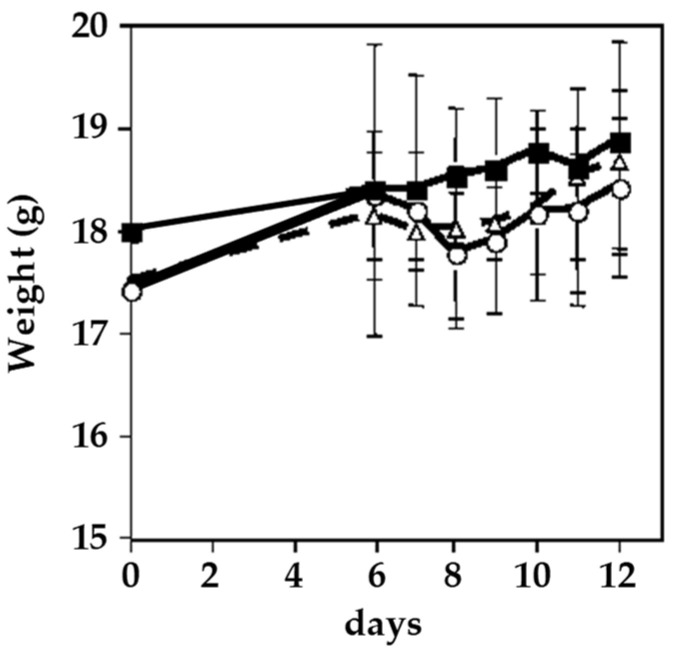
Effect of Phx-3 on the body weight of mice transplanted with B16 cells (○: Phx-3 △: positive control ■: negative control). Source: Miyano-Kurosaki et al. [51] by permission from *Biol. Pharm. Bull*.

Figure 6 shows how no weight loss was observed in mice after the treatment with Phx-3. In addition, no pathological changes were observed in the liver and kidneys, or in serum levels of relevant blood biochemical parameters.

In a subsequent study [75], it was found that Phx-3 inhibits the expression of tyrosinase (rate-limiting melanogenic enzyme), as well as the transcription factor that regulates its expression.

From the results mentioned in this section, Phx-3 could be proposed as suitable candidate drug for the treatment or prevention of melanoma, including its cosmetic use to treat hyperpigmentation and to prevent melanogenesis.

## 4. Activity of Aminophenoxazinones on Other Cell Lines

### 4.1. 2-Aminophenoxazine-3-one (Phx-3)

First of all, we should highlight the study that Che et al. carried out to evaluate the cytotoxicity of Phx-3 on different types of cancer cells, such as MCF-7, A431, KCP-4, A549, KLM-1, MIA PaCa-2, ACHN, LoVo-1, U251MG and Y-79 lines [57]. In the case of ACHN (renal carcinoma), Liu et al. had already reported the modest cytotoxic activity generated by Phx-3, as well as for a methylated and acetate derivative of this aminophenoxazinone [76]. Looking at IC_50_ values, the sensitivity of the mentioned cancer cell lines after 72 h of treatment with Phx-3 showed how almost all of them were vulnerable to Phx-3 at 10 µM (IC_50_ lower than 8 µM). KLM-1, Lovo-1 and Y79 lines were the exceptions (IC_50_ close to 20 µM). The normal cell lines HEL (embryonic pulmonary fibroblast) and HUVEC (umbilical cord) were also tested, being obtained high IC_50_ values (over 50 µM and 16 µM, respectively), which indicates that these cells are less sensitive to Phx-3 than cancer cells.

**Figure 7:**
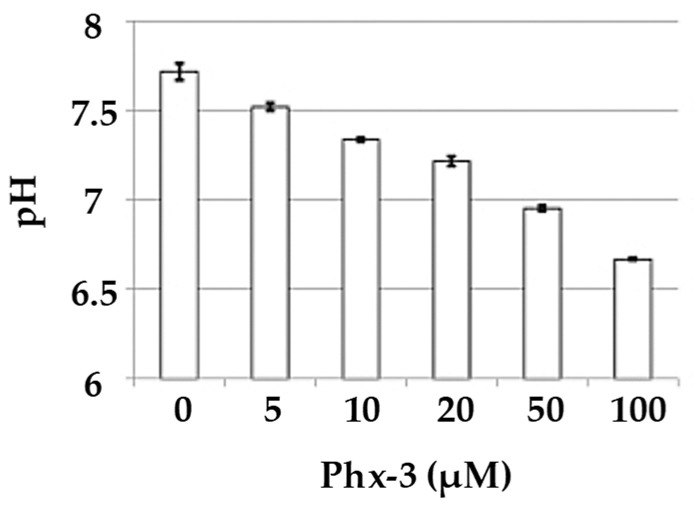
Changes in the pHi of MCF-7 cells treated with different concentrations of Phx-3 Source: reproduced from Che et al. [57] with permission from *Proc. Japan Acad. Ser. B*.

Regarding the pHi decrease, significant results were obtained for all cancer cell lines (reduction of 0.22–1.00 units at 20 µM, and 0.64–1.20 units at 100 µM), being comparable to those of the normal lines. The decrease was proved dose-dependent for MCF-7 (breast cancer) and A431 (skin cancer) cell lines for 30 min and ranging between 0 and 100 µM (Figure 7). Therefore, it can be concluded that the use of Phx-3 causes a drastic acidification of cancer cells, which in turn induces their apoptosis [77]. Phx-3 would be a suitable drug for the treatment of cancer, as it causes drastic decreases in pHi (by more than 0.6), and induces apoptosis and cytotoxic effects on cancer cells without significant adverse effects.

**Figure 8:**
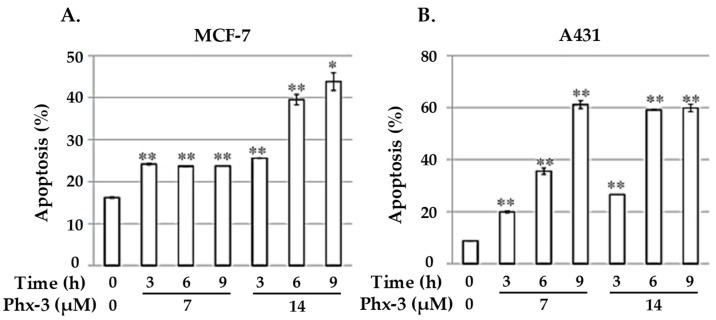
Proapoptotic effects of Phx-3 on (**A**) MCF-7 and (**B**) A431 cells. Reproduced from Che et al. [57] with permission of *Proc. Japan Acad., Ser. B.* * *p* < 0.01, ** *p* < 0.001.

MCF-7 and A431 lines were further evaluated to provide conclusions of the mechanism of action of Phx-3, attending at the reduction of the mitochondrial membrane potential (first and irreversible step towards apoptosis). Figure 8 shows how the population of both MCF-7 and A431 lines, determined as their decrease in mitochondrial potential, increased as a direct function of both time and concentration of Phx-3. According to these data, and considering the reduction of the pHi value in both cell lines, it can be concluded that the apoptosis of MCF-7 and A431 cells could be preceded by the early acidification caused by Phx-3.

In other study, the cytotoxic and pro-apoptotic effects of Phx-3 on hepatocellular carcinoma dRLh-84 (rat) and HepG2 (human) cell lines, and the normal hepatocellular RLN-10 (rat) cell line have been studied [61]. Phx-3 reduced the number of viable cells in the three lines by a dose-dependent degree, being 2 µM enough to induce apoptosis by nuclear condensation and cell shrinkage. Moreover, Phx-3 combined with 2-deoxyglucose significantly enhanced apoptosis, but, on the other hand, some adverse effects were observed on normal liver cells. More recently, the combined treatment of Phx-3 with sorafenib was demonstrated to suppress the formation of hepatocellular carcinoma on in vivo studies. Phx-3 was reported as suppressor of the expression of GRP78, target protein directly related with different cancer cell lines, in HepG2 cells [78]. It must be noted that Phx-3 is named as questiomycin A in this last article.

The list of cancer lines with cell whose growth can be inhibited by Phx-3 may be completed with HeLa (cervical cancer, IC_50_ = 12.09 ± 3.29 µM) [37], U266 (myeloma), HL-60 (acute myeloid leukemia) and A549 (lung adenocarcinoma) [71] lines. The study of Moriya et al. (2011) was also focused on the pro-apoptotic transcription factor CHOP, being suggested that the regulation of its expression could represent a major target for treatments. So, in the case of U266, the activity of Phx-3 was enhanced by its combined application together with an inhibitor of NF-κB, a transcription factor related to the inhibition of CHOP.

### 4.2. Amino-4,4α-dihydro-4α,7-dimethyl-3H-phenoxazin-3-one (Phx-1)

Phx-1 (Figure 3) is an aminophenoxazinone with anticancer activity, obtained from the reaction of 2-amino-5-methylphenol with bovine hemoglobin [79]. The in vitro studies that have been conducted on this compound are similar to those previously described for Phx-3 [57].

Regarding the pHi reduction in MCF-7 and A431 cancer cell lines, similar or rather close results as those from Phx-3 were observed at the lowest concentrations (5–10 µM) were applied. However, Phx-1 proved to be significantly less active than Phx-3 at 50 µM, and concentration had to be increased up to 100 µM for any major differences to appear in the A431 line.

The study of Phx-1 on diverse cell lines (the same previously mentioned in the first paragraph of Section 4.1), revealed that this compound achieves significant pHi reductions, but not as high as those attained by Phx-3. Thus, Phx-1 reached 0.01–0.16 at 20 µM and 0.11–0.59 at 100 µM, whereas Phx-3 achieved 0.22–1.00 at 20 µM and 0.64–1.20 at 100 µM. In both cases, the normal cell lines tested (HEL and HUVEC) suffered an equally significant decrease in pHi, comparable to that suffered by cancer cells.

The data obtained in relation to the cytotoxic effects of Phx-1, compared with those of Phx-3, revealed that the cytotoxicity generated by Phx-1 on all the tested cell lines was much lower (the most sensitive cancer line was MCF-7). These results are in agreement with the pHi changes undergone by the cells treated either Phx-1 or Phx-3 that have been summarized in the previous paragraph. This could represent a certain advantage regarding cancer treatment, since healthy cells would suffer a lesser damage.

Although the IC_50_ value of Phx-1 for Y-79 cells (retinoblastoma, eye cancer) is the highest, previous studies [80] showed in vivo antitumor effects in cells transplanted into mice whose strain suffers from a genetic mutation that causes the deterioration or lack of the thymus (organ where T cells mature). Phx-1 induced in vivo apoptosis to Y-79 in mice without any type of adverse effect even at high doses. These favorable signs make of Phx-1 a suitable candidate for the development of drugs for the treatment of retinoblastoma. In general, although Phx-1 is not highly effective, it could facilitate the induction of apoptosis and exert a cytotoxic effect on cancer cells.

It is worth highlighting that Phx-1 inhibited the proliferation and induced the apoptosis of diverse human leukemia cell lines (K562, HL-60 and HAL-01) in a dose-dependent manner. This study also proved that Phx-1 reduced in vivo the tumor growth rate in mice, whereas just few adverse effects were found on weight loss and white blood cell [81].

Once confirmed the ability of Phx-1 and Phx-3 to induce the apoptosis of diverse cancer cell lines, Tabuchi et al. studied the effects of these compounds to induce apoptotic cell death in human neutrophils, as this kind of cells are related with the mitochondrial depolarization and reduction of pHi. Both aminophenoxazines caused apoptosis or the loss of the morphology of neutrophils, while lymphocytes and monocytes did not undergo this process. These results would suggest that Phx-1 and Phx-3 are specific drugs to induce apoptotic cell death of neutrophils, being potential preventive anti-inflammatory drugs [58].

The antitumor activity of Phx-1 and Phx-3 on NB-1 (neuroblastoma cell line) was demonstrated by another study. It was thereby confirmed that both aminophenoxazinones induced apoptosis and necrosis, being the IC_50_ value of Phx-3 much lower (0.5 µM vs. 20 µM) [82].

From the structural point of view, the differences in the bioactivity exhibited by Phx-1 and Phx-3 would be associated to the methyl group in Phx-1, even this premise is still to be confirmed. Molecular dynamics simulations could cast some light on the solubility of these compounds in cell membranes, which would allow to determine how much their structural differences affect cross-membrane processes and, thereby, explain the differences in their activity levels. In this line, certain recent studies have proven that the replacement of hydroxyl groups by fluorinated esters (in the eudesmanolide structure), improves the cytotoxicity of these compounds to cancer cells (HeLa, cervical cancer) [83].

### 4.3. Phenoxazine-Indole Conjugates

**Figure 9:**
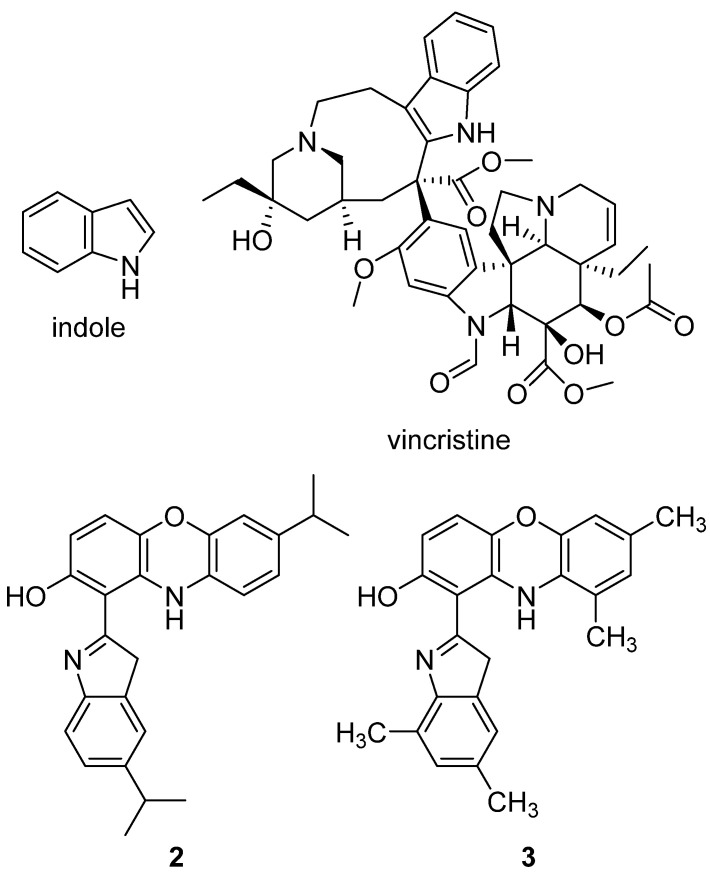
Structures of indole, the anticancer drug vincristine and the most active indole derivatives.

Recent studies have tested the activity of different derivatives with an indole group on different cancer cell lines. Indole groups are heterocyclic compounds formed by a benzene atom linked to a pyrrole compound, that have a pair of free electrons in the nitrogen atom of their aromatic ring. They are fairly common components in perfumes, drug candidates and hormones (like melatonin) [84]. Some natural indole alkaloids, such as vincristine (Figure 9), have been approved in the USA by the Food and Drug Administration (FDA) as antitumor drugs [85].

Taking into account the potential pharmacological effects of indoles, as well as those already recognized of phenoxazines and the synergistic effects exhibited by some pharmacophoric hybrids [86], Nunewar et al. [87] suggested that certain hybrids formed by two of these groups would possess some proliferative effect by operating as DNA exchangers. The general proposed mechanism consists of a first transfer of the molecule to the hydrophobic space between two adjacent DNA base pairs. As consequence, DNA would undergo conformational changes, in order for the molecule to accommodate itself between the base pairs. The resulting complex prevents DNA replication, which would lead to the death of the cell, being these results especially useful for the treatment of rapidly growing cancer cells.

Therefore, the cytotoxicity of certain indol derivatives was tested by Nunewar et al. on A549 (lung), MG-63 (bone), BT-474 (breast), Hep G2 (liver) and HCT116 (colon) human cancer cell lines, along with a normal line of lung epithelial tissue (L-132). All the derivatives tested showed remarkable IC_50_ values. The most inhibited cell line was A549, by the derivative **2** (IC_50_ = 3.71 ± 0.57 µM), characterized by two isopropyl substituents (Figure 9), and followed by compound **3** (IC_50_ = 4.43 ± 0.64 µM). In addition, **2** was the only derivative with the previously explained DNA intercalation capacity. These results confirm that hybrid compounds are a promising alternative in the search for new anticancer drugs.

### 4.4. Pyridophenoxazinone Derivatives Conjugated to L-Lysine

**Figure 10:**
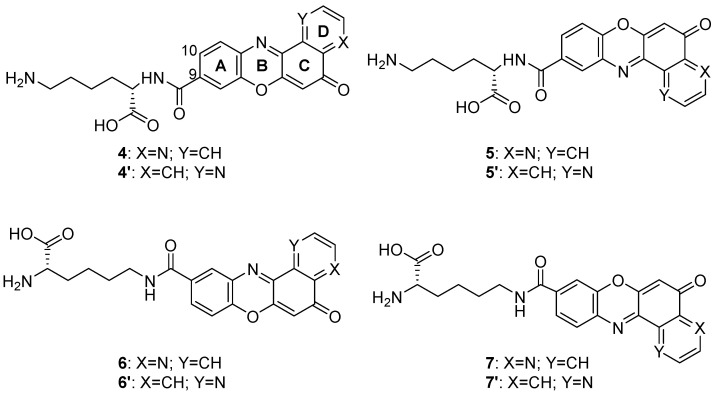
Structure of the pyridophenoxazinone derivatives conjugated to L-lysine.

Also in 2020 a number of studies were conducted on a series of pyridophenoxazinones (which possess intercalating capacity, and for generating free radicals that induce cell death by oxidative-stress) conjugated with the amino acid L-lysine, which were designed and synthesized with the aim of developing novel drug compounds with anticancer potential [88]. Similarly to those described in the previous section, these studies focused on the ability of the compounds to intercalate between the base pairs of nucleic acids. Thus, synthetized derivatives contained a basic side chain of L-lysine in the positions 9 or 10, and the *N*-terminal group of L-lysine was linked to the chromophore through an amide bond (Figure 10).

Products **4**–**7** and **4**′–**7**′ were tested on cancer cell lines of leukemia (CCRF-CEM, CCRF-SB and MT-4), colon (HT-29), breast cancer (MCF-7), cervical cancer (HeLa), papillary renal cell carcinoma (ACHN), and melanoma (SKMEL-28 and G-361). All the products inhibited the proliferation of a panel of human liquid and solid neoplastic cell lines, the latter being more sensitive to antiproliferative effects (only compound **7** showed similar activity in both states). The IC_50_ values of the derivatives with the L-lysine side chain attached to C-10 were higher than their corresponding derivative functionalized at C-9. The IC_50_ values of **4**–**7** were comparable to that of AMD and even lower than those of Doxo and VP-16 (commercial drugs). It is worth highlighting the lowest values of IC_50_, all in the range of 0.001–0.007 µM, achieved by **4** and **7** for HT-29, SKMEL-28, MCF-7 and G-361 lines. The authors proved that both compounds are strong DNA intercalators, and possess the capacity to selectively target the topoisomerase Topo IIα.

The difference in activity between the two series of derivatives was related to structure-activity correlations. Thus, the position of the nitrogen atom in D-ring would play an important role in the antiproliferative activity, whereas the position of the L-lysine side chain and the type of amine groups affect the cytotoxic activity both between the two series and within each series.

All the mentioned properties indicate that this new series of pyridophenoxazinones conjugated to L-lysine have a great antitumor therapeutic potential. Products **4** and **7** in particular, provide really interesting opportunities for the development of new DNA-targeting anticancer drugs.

## 5. Antibacterial and Antifungal Activities

The emergence of resistant bacteria and its impact on health, together with the inappropriate use of antibiotics, has become a pressing problem for patient safety. Infectious diseases are one of the main causes of global morbidity and mortality, especially in developing countries.

The study of Shimizu et al. [89] proved that Phx-1, Phx-2 and Phx-3 (Figure 3) can exhibit antibacterial activity. These three molecules were tested on seven non-tuberculous mycobacteria species, being noticeably active against *Mycobacterium scrofulaceum* attending to minimum inhibitory concentration (MIC) values obtained (1.4–2.8 µg/mL). Phx-3 also inhibited *Mycobacterium marinum* and *Mycobacterium intracellulare*, with slightly higher MIC values, whereas *Mycobacterium kansasii* showed the best inhibition levels for Phx-1 and Phx-2 (Table 1). These results may suggest that the aminophenoxazinones Phx-1, Phx-2 and Phx-3 could be used for the treatment of non-tuberculous mycobacteria infections. The sensitivity to these compounds is related to the characteristics of the cell wall of the strains. Cell wall structure is composed of an innermost electron-dense layer, an electron transparent zone at the intermediate layer and the outermost electron-dense layer (composed of various lipoglycans, free polysaccharides, glycolides and phospholipids) [90].

In the case of Phx-3, high antituberculosis activity levels have been also reported (MIC = 3.91 µM) [56], as well as promising antiinflammatory and immunoregulatory activities in in vivo assays in mice [59]. These last conclusions were related with the inhibition of the production of nitric oxide and prostaglandin E2, both achieved with low IC_50_ values (1.5 and 0.27 µM, respectively). Phx-3 was suggested for the treatment of T cell-mediated autoimmune diseases, and for those chronic-inflammatory diseases induced by bacteria.

### 5.1. Antibacterial Activity on Genera Helicobacter

*Helicobacter pylori*, which affects approximately 50% of the world population, is a causative agent of chronic gastroduodenal ulcer and, possibly, gastric cancer. The triple therapy that comprises amoxicillin, clarithromycin (CAM) and a proton pump inhibitor, has proven to be effective to eradicate this bacterium. However, resistance is a common issue that appears when applied to the control of bacteria like *H. pylori*. As an example, the recovery rate when this triple therapy treatment is applied in Japan is as low as 20% [91]. This loss of effectiveness caused by resistance phenomena is therefore a serious concern with regard to the eradication of this type of infection [92].

This resistance issue has brought about the development of alternative drugs over the last decade, being Phx-3 one of the drugs of interest for this purpose [93]. Phx-3 was, therefore, tested against standard strains of *H. pylori* (including 43,504 and ATCC 43579) and against three clinical strains (TK 1029, 1047 and 1402). According to the results regarding cell-mediated immunity (CMI), none of the strains, including TK 1029, which has proven to be resistant to the triple therapy, grow in the presence of Phx-3 at concentrations of 1 µg/mL or higher. After 48 h, the bacterial populations were below their detection limit. Phx-3 has also been tested against *H. musterae* (ATCC 43772), and 0.5 µg/mL CMI value was reported.

Inhibitors of *H. pylori* have the capacity to implement certain morphological changes [94]. Such changes that take place after the compound has been administered need to be evaluated. Thus, cells in ATCC 43,504 strains were subjected to a culture of Phx-3 (6 µg/mL) for 6 hours, after which, severe morphological changes were revealed by scanning electron microscopy, namely the detachment of outer membranes, which might be the mechanism responsible for the rapid bactericidal activity of TG44. In the case of Phx-1 and Phx-2, no activity was found against *H. pylori* and *H. mustelae* strains.

Information provided herein would be quite relevant for the development of new drugs for the control of H. pylori based on Phx-3. Some reports associate *H. pylori* infections to gastric lymphomas and adenocarcinomas (currently known as type 1 carcinogens) [95]. Therefore, the control of this bacterium also contributes to the prevention of certain types of cancers.

### 5.2. Other Antibacterial and Antifungal Activities

Similarly to the genera *Helicobacter*, the appearance of certain antimicrobial resistance represents a menace to the prevention and treatment of infections caused by bacteria, parasites, viruses and fungi. The diseases associated to these organisms are the third leading cause of death in developed countries [96]. For instance, the virulence of *Fusarium* spp. (fungi) in plants is conditioned by the resistance generated by the detoxification of certain drugs, like benzoxazolinones BOA and MBOA (plant defense compounds). This process was found to be catalyzed by the fungal γ-lactamase enzyme encoded by FDB1 locus [97]. Therefore, new strategies or compounds that are not affected by this resistance need to be developed.

Phx-3 has shown high levels of toxicity to the species *Fusarium verticilloides* [98]. Tested at a 50 µM dose, it is also active against *Bacillus subtilis* and *Staphylococcus aureus*, as well as on the fungus *Candida albicans*, whereas no activity against *Escherichia coli* was observed. The derivative hydroxylated at C-6 was active for the two first species, with slightly better results [99]. We would like to recommend to all those who intend to conduct further studies on the activity of Phx-3 against *S. aureus* the article by Tse et al. [100]. Later on, a silver(I) complex of Phx-3 (the first reported metal complex of this compound) showed an improvement of the activity against *S. aureus*, that was attributed to the release of silver ions into the cells after getting dissociated from the complex. Like Phx-3, this complex was inactive against *E. coli*, which was probably explained by the obstacle posed by the double layer cell wall that characterizes gram-negative species [23].

Chandrananimycin A (Figure 2) and B are natural products related to Phx-3, characterized by the presence of an amide function instead of the common exocyclic amine group. Both have shown inhibition activity on *Mucor miehei* and, in the case of the first, also on *B. subtilis* [101]. In the same study chandrananimycin C was also evaluated, being active on the same species (increasing almost to the double the inhibition zones) together with *S. aureus*, *C. albicans*, *Chlorella vulgaris*, *Chlorella sorokiniana* and *Scenedesmus suspicatus*. The better activity of chandrananimycin C in the concentration tested (20 µM) would be related to its structure, which presents the scaffold of chandrananimycin B with an additional fourth ring (cyclic amine). None of the three compounds were active *on E. coli*.

The natural product NHP (Figure 4), in addition to the cytotoxicity on cancer lines previously mentioned in Section 3.1, has shown antifungal activity against *C. albicans* (MIC = 64 µg/mL) [60].

**Figure 11:**
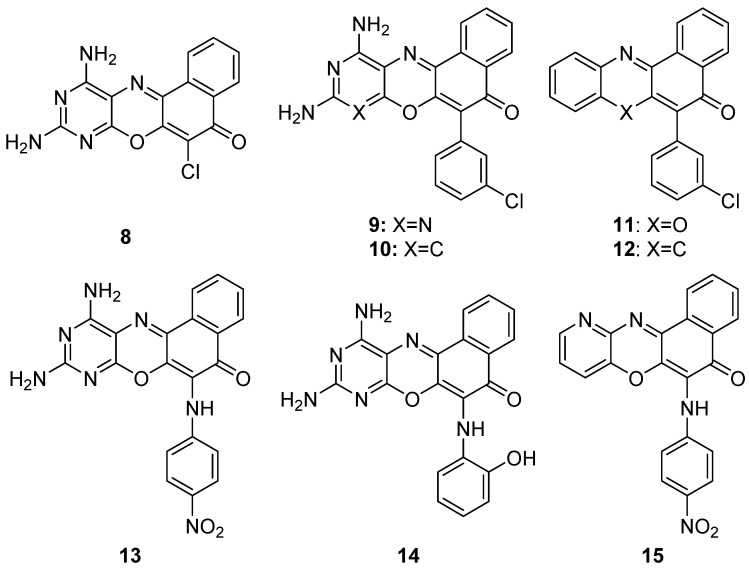
Compound **8** and derivatives obtained by arylation using boronic acids (**9**–**12**) or by amination using anilines (**13**–**15**).

Recent studies [102] evaluated the antimicrobial activity of 8 derivatives (**8**–**15**) of phenoxazines against the bacteria *Listeria ivanovii*, *E. coli*, *S. aureus* and *Klebsiellapneumoniae* spp., and against the fungi *C. albicans* and *Aspergillusniger* spp. For the synthesis of the compounds, in a first stage, **5** was synthesized, then, the rest of compounds were obtained by arylation (compounds **9**–**12**) or amination (compounds **13**–**15**) reactions of **8** (Figure 11).

The agar well diffusion method was employed for the sensitivity study of **8**–**15**. All the compounds showed a considerable activity against *L. ivanovii*, except compound **12**. Only half of the compounds (**8**, **9**, **14** and **15**) exhibited activity against *E. coli*, which was more selective. In the case of *S. aureus*, *K. pneumoniae*, *C. albicans* and *A. niger*, it should be highlighted that only compound **9** presented some activity against all these species. It should also be noted that compound **8** (scaffold of **9**–**15**) showed sensitivity to all the organisms except *S. aureus* and *L. ivanovii*, both of which are gram-positive bacteria. Therefore, it could be concluded, with regard to molecular structures, that the compounds obtained either through the arylation or amination of compound **8** causes the inactivation of the molecule on fungi, but exhibits an increased activity when applied to gram-positive bacteria.

The data obtained from the experiments show how most of the compounds are active at low concentrations. The best minimum active concentration observed was for derivatives **11** and **15** on *L. ivanonii* (14.42 µg/mL), being a significantly lower value than that of the reference drug (21.30 µg/mL). This higher activity than currently commercialized drugs, let us think that new derivatives could be developed to a more efficient control of microorganisms.

**Figure 12:**
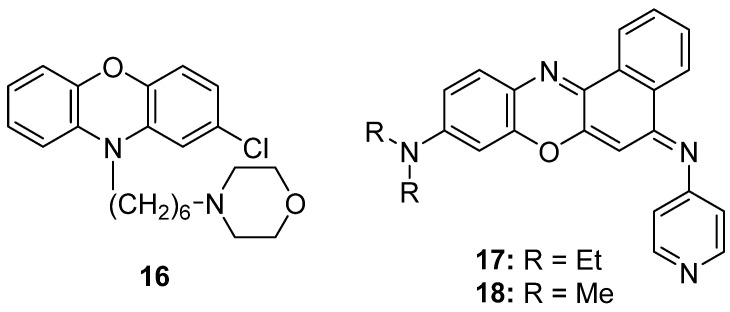
Phenoxazin derivatives with antibacterial (**16**) and antimalarial (**17** and **18**) activity.

Sridhar et al. [103] reported the evaluation of a family of chlorinated phenoxazine derivatives characterized by the presence of a tertiary amine at the second ring. Each product presents a specific moiety bonded to this nitrogen atom. Four of these products showed to be significantly active on *Klebsialla* sp., *Bacillus* sp., *Proteus vulgaris*, *Salmonella* sp., *Shigella flexneri*, *S. aureus*, *Streptococcus pneumoniae* and *Vibrio cholerae*, with compound **16** being remarkable (Figure 12).

The final study presented in this section will be the finding of new chromogen aminophenoxazinone derivatives with applicability for the detection of *Pseudomonas aeruginosa* (a pathogenic Gram-negative bacterium) by detecting β-alanyl aminopeptidase activity in clinical samples [104]. This family of compounds is characterized by the presence of a β-alanyl moiety bonded to the first ring of aminophenoxazinones via an amide bond. In the article, it is provided the results of a broad screening test of the compounds on diverse bacterial species of both gram categories.

## 6. Other Activities

### 6.1. Antiviral Activity

In vivo antiviral studies of aminophenoxazinones Phx-1, Phx-2 and Phx-3 in mice allowed to propose the three compounds as potential drugs for preventing the replication of HSV and the aggravation of lesions that these viruses cause. Their activity on herpes simplex virus type-1 (HSV-1) was highlighted, whereas Phx-2 was the only that showed inhibitory effects against HSV-2 (moderately). Another point of interest regarding HSV-2 was the improvement of the survival rates after the treatment with Phx-1, Phx-2 or Phx-3 (without treatment, all the infected did not survived) [105]. Phx-2 also inactivates human cytomegalovirus (HCMV) in vitro, being measured a markedly better value of selectivity index [106]. The inactivity of Phx-3 on H1N1 and H3N2 influenza viruses has been reported [56].

The antiviral potential of the three compounds is also promising for the human T-lymphotropic virus type-1 (HTLV-1), which causes adult T-cell leukemia (ATL). Phx-1, Phx-2 and Phx-3 prevent the cell-to-cell transmission of this virus in model cell lines, as well as the induction of apoptosis in MT-1 cells in doses of 10 μg/mL [107]. The same study suggested that this apoptosis process occurs by post-translational events like the depolarization of mitochondria, as the aminophenozaxizones activated the enzyme caspase-3.

Recently, Phx-3 was computationally studied by docking to evaluate its pharmacokinetic related with the inhibition against MMP9 enzymes, whose overexpression is caused by the infection of hepatitis B virus [108]. As result, Phx-3 would possess key properties under the acceptable range, however, it was not among the most potent compounds evaluated in this study.

### 6.2. Antiparasitic Activity

Malaria is a parasitic disease that causes high fevers, chills, flu-like symptoms and anemia. It is caused by a parasite and is transmitted to humans through the bite of infected *Anopheles* mosquitoes [109].

Ge et al. [110] evaluated the antimalarial activity of various phenoxazin derivatives. The best results were obtained from the derivative functionalized with a 4-aminopyridine group (**17**, Figure 12). The activity level measured for **18** should also be highlighted. However, this compound presented the disadvantage of its shorter life; probably due to the shorter chains of the tertiary amine.

Compound **17** was considered a suitable candidate for the development of antimalarial drugs. In the first place, it exhibited an IC_50_ of 7.6 nM it against *Plasmodium falciparum* as well as a noticeable higher selectivity when compared to the other synthesized derivatives. Moreover, the in vivo experiments that were carried out on mice infected with *Plasmodium berghei* (the parasitic species that causes malaria in some rodents) achieved the cure after three daily doses for three consecutive days.

**Figure 13:**
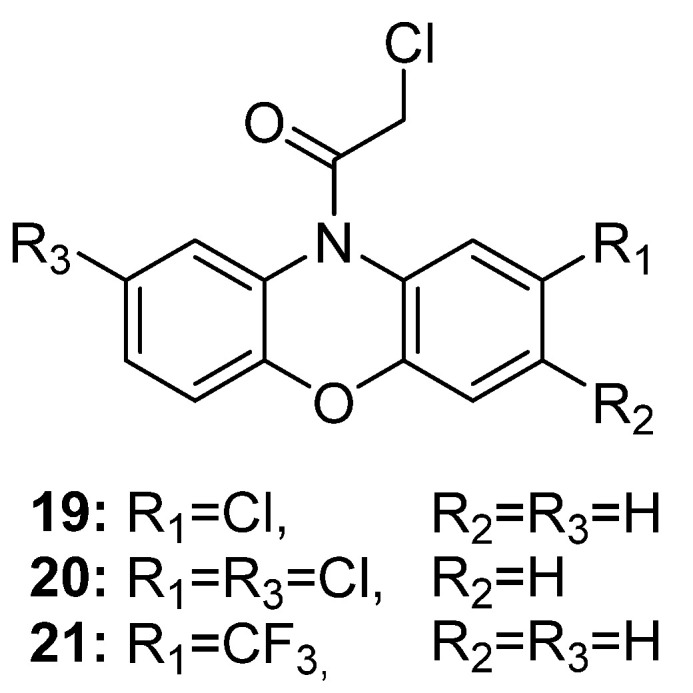
Compounds that have exhibited some interesting potential to treat *L. major* infections.

*Leishmania major* is a parasitic species that causes cutaneous leishmaniasis, a tropical disease that generates a large number of adverse effects on human health, mainly affecting the skin and mucous membranes. Human infections occur after the bite of sand fly (*Phlebotomus*). A number of haloacetamines derivated from *N*-substituted phenoxazines, as well as other related structures such as phenothiazines, have been studied through in vitro essays [111]. The haloacetamide derivatives proved to be suitable to inhibit *L. major*. The lowest IC_50_ values were obtained by the products shown in Figure 13: 7.6 µM (**19**), 9.5 µM (**20**) and 9.6 µM (**21**).

## 7. Conclusions

Our compendium of relevant studies from the literature leads us to conclude that aminophenoxazinones possess a number of promising properties like anticarcinogenic, antifungal, antiparasitic, antibacterial or antimicrobial activities. Aminophenoxazinones are degradation products that present really interesting pharmacological properties, selectivity and minimal toxicity that make them suitable for the treatment of certain current and relevant diseases as explained in this review. Phx-1, Phx-2 and Phx-3 are the most studied aminophenoxazinones in the literature, especially Phx-3, whose activities and modes of action have been detailed herein. In the case of their cytotoxicity against cancer cell lines, Phx-3 stands out as promising anticancer drug, due to the capacity to decrease the pHi in cancer cells and inhibit the proliferation of cell lines like the related to gastric and colon cancer, glioblastoma or melanoma, among others. The activity of Phx-3 has been compared to that of anticancer drugs like camptothecin, known for its side effects. In this context, in vivo studies have shown that Phx-1 and Phx-3 cause low or null adverse effects, which indicates that they would be less aggressive drugs. Regarding their antibacterial and antimicrobial activities, Phx-1, Phx-2 and Phx-3 have proven to have the desirable characteristics that would allow certain drug resistance issues to be overcome. Some non-tuberculous mycobacteria, *H. pylori* or *F. verticilloides* are examples of high sensitive species to these aminophenoxazinones. Moreover, it should be remarked their activity against herpes simplex virus type-1 (HSV-1) and human T-lymphotropic virus type-1 (HTLV-1). Remarkable antiproliferative capacity have been proven for some structural derivatives, such as those conjugated to indole groups **2** and **3** (especially for lung adenocarcinoma), the pyridophenoxazinones **4** and **7** (for melanoma, colon and breast cancer), compounds **8**, **9**, **11**, **15** and **16** (antibacterial or antifungal), **17** (antimalarial) and **19**–**21** (against *Leishmania major*).

Therefore, the active compounds mentioned herein could be considered for the development or improvement of therapeutic treatments, from the perspective of the activity levels. This conclusion supports the research studies that are currently being conducted on this family of compounds. In the authors’ opinion, it is necessary the seek and evaluate new derivatives that could allow one to define the structural features that lead to the effects observed in the different types of assays. Even though different in vivo studies have been presented in this review, we consider that the proportion of this type of assays should increase over the next years, which would bring firm conclusions for the future use of aminophenoxazinones and related compounds as drugs.

## Figures and Tables

**Table 1:** Minimum inhibitory concentration values (µg/mL) obtained by Shimizu et al. [89] for the compounds Phx-1, Phx-2 and Phx-3 against different species of the genera *Mycobacterium*.

Species	Phx-1	Phx-2	Phx-3
*M. scrofulaceum*	2.8	1.4	2.8
*M. marinum*	>45	>45	11.3
*M. intracellulare*	>45	>45	5.6
*M. kansasii*	22.5	11.3	>45
*M. tuberculosis*, *M. fortuitum* and *M. smegmatis*	>45	>45	>45

## Data Availability

The data presented in this study are available on request from the corresponding author.
